# Oral microbiome alterations in individuals with type 1 diabetes: a systematic review

**DOI:** 10.1007/s00784-026-07205-y

**Published:** 2026-10-09

**Authors:** Aspasia Pachiou, Mariana Damanaki, Pune Nina Paqué, Christos Rahiotis, Eftychia Pappa

**Affiliations:** 1 Clinic of Reconstructive Dentistry, Center for Dental Medicine, University of Zurich, Plattenstrasse 11, Zurich, CH-8032 Switzerland; 2 Department of Prosthodontics, School of Dentistry, Aristotle University of Thessaloniki, Thessaloniki, Greece; 3 Department of Operative Dentistry, School of Dentistry, University of Athens, Athens, Greece

**Keywords:** Type 1 diabetes mellitus, Glycemic control, Oral microbiome, Oral dysbiosis, Periodontal pathogens, Candida

## Abstract

**Objectives:**

To systematically evaluate associations between glycemic control and oral microbial findings in individuals with type 1 diabetes mellitus (T1DM), distinguishing targeted detection of selected taxa from comprehensive oral microbiome profiling.

**Materials and methods:**

A systematic search of PubMed, Embase, and Scopus was conducted from database inception to June 2025 in accordance with the PRISMA statement. The protocol was prospectively registered in PROSPERO. Human studies investigating the relationship between glycemic control and the oral microbiome in individuals with T1DM were eligible for inclusion. Two reviewers independently performed study selection, data extraction, and risk of bias assessment. Due to substantial methodological heterogeneity, findings were synthesized qualitatively.

**Results:**

Forty-two studies met the eligibility criteria. Most were cross-sectional and assessed glycemic control using glycated hemoglobin (HbA1c), with heterogeneous thresholds. Saliva and subgingival plaque were the most frequently sampled oral sites. Targeted studies commonly reported associations between poorer glycemic control and selected periodontal pathogens or oral Candida carriage, whereas findings for cariogenic microorganisms were inconsistent. Only four studies used comprehensive culture-independent community profiling; these studies reported differences in community composition and/or diversity, but the small number and methodological heterogeneity preclude a uniform conclusion regarding the direction of diversity change.

**Conclusions:**

Poorer glycemic control is associated with selected oral microbial alterations in individuals with T1DM, particularly in periodontal and Candida-focused studies. However, the predominance of targeted assays, cross-sectional designs, and substantial methodological heterogeneity limits conclusions about whole-community oral microbiome dysbiosis or causality. Well-designed longitudinal studies using standardized clinical phenotyping and comprehensive microbiome profiling are needed.

**Clinical relevance:**

Poor glycemic control may coexist with adverse oral microbial and clinical findings in individuals with T1DM. These associations support attention to established preventive dental care and periodontal assessment as part of multidisciplinary diabetes care; however, oral microbiome monitoring is not yet validated as a clinical biomarker of glycemic status or oral disease risk.

**Supplementary Information:**

The online version contains supplementary material available at https://doi.org/10.1007/s00784-026-07205-y.

## Introduction

Type 1 diabetes mellitus (T1DM; insulin-dependent diabetes) is a chronic autoimmune disease characterised by absolute insulin deficiency and lifelong need for exogenous insulin [1]. Despite major advances in insulin delivery and glucose monitoring, many individuals experience periods of suboptimal glycemic control, which contributes to microvascular and macrovascular complications and alters host immune and inflammatory responses [2, 3]. These systemic effects are also clinically relevant in the oral cavity, where diabetes is consistently linked to increased susceptibility to periodontal inflammation and impaired wound healing [4].

Periodontitis and diabetes are now widely recognised as having a bidirectional relationship: diabetes increases the risk and severity of periodontitis, while periodontal inflammation can adversely affect glycemic control and contribute to systemic inflammatory burden [5]. A landmark joint workshop consensus report highlighted a dose-dependent association between periodontitis severity and diabetes-related complications, emphasizing the need for integrated medical–dental management [6]. In people with T1DM specifically, evidence also supports a meaningful association with periodontal disease and related inflammatory markers, although heterogeneity in study designs and outcomes remains a limitation [7, 8].

A potential biological link between glycemic control and oral disease is the oral microbiome [9]. The oral cavity hosts complex microbial communities (supragingival plaque, subgingival plaque, saliva, and gingival crevicular fluid niches) that can shift from symbiosis to dysbiosis under inflammatory and metabolic stress [10]. Hyperglycemia may influence microbial ecology directly (via glucose availability in oral fluids) and indirectly by modifying host defenses, tissue glycation, oxidative stress, and cytokine signaling [11]. In diabetic conditions, increased advanced glycation end-products (AGEs) and inflammatory mediators may shape periodontal tissues and the subgingival environment, potentially favoring dysbiotic communities [12].

Emerging sequencing-based studies in children and adolescents with T1DM report measurable differences in salivary or dental biofilm microbiota compared with non-diabetic controls, and several investigations suggest that glycemic control (commonly assessed by glycated hemoglobin HbA1c) may correlate with microbial patterns and early periodontal risk markers [13, 14]. However, the available evidence is scattered across heterogeneous sampling sites, age groups, disease duration, and periodontal status, and studies vary widely in how “regulated” versus “unregulated” diabetes is defined, such as different HbA1c thresholds, which complicates synthesis and clinical interpretation.

Accordingly, this systematic review evaluates the association between glycemic control in T1DM and oral microbial findings. Because most included studies used culture-based or targeted molecular methods, the term “oral microbiome” is used to refer to the broader ecological concept, while describing findings from targeted assays as changes in selected microbial taxa. The review also examines whether associations differ by oral niche, clinical oral disease status, age, and methodological approach, and identifies gaps in sampling, comprehensive sequencing, and confounder control.

## Materials & methods

### Protocol registration and reporting guidelines

This systematic review was conducted in accordance with the Preferred Reporting Items for Systematic Reviews and Meta-Analyses (PRISMA 2020) guidelines [15]. The review protocol was prospectively registered in the PROSPERO database (registration ID: CRD420251057230). All methods were predefined and followed the registered protocol.

### Review question and objectives

The objective of this systematic review was to evaluate associations between glycemic control/status and oral microbial findings in individuals with T1DM. The focused review question was: “What associations have been reported between glycemic control/status and oral microbial composition, diversity, or selected microbial taxa in individuals with T1DM?”

### Eligibility criteria

Studies were eligible for inclusion if they involved human participants diagnosed with type 1 diabetes mellitus (T1DM), irrespective of age, sex, or ethnicity. Older publications frequently used the historical term “insulin-dependent diabetes mellitus (IDDM)”; in this review, such studies were eligible only when the study population was described as having T1DM/juvenile-onset autoimmune diabetes, and insulin treatment alone was not considered sufficient to establish T1DM. The exposure of interest was glycemic control/status, assessed using objective measures such as HbA1c, blood glucose measures, or longitudinal clinical indicators of diabetes control. No explicit comparator group was required for inclusion. Eligible studies were required to investigate oral microbial composition, diversity, or selected microbial taxa in relation to T1DM and/or glycemic control. Both randomized and non-randomized study designs were considered, including observational and interventional studies. Studies conducted in any geographical location or clinical setting were eligible, provided that the methodology and setting were clearly described. Only studies published in English and in peer-reviewed journals were included. Studies were excluded if they involved preclinical or in vitro models, focused on non–insulin-dependent diabetes mellitus, gestational diabetes, or prediabetes, or did not assess oral microbiome outcomes. Case reports, narrative reviews, editorials, letters, conference abstracts, and studies lacking objective assessment of glycemic control were also excluded.

### Information sources and search strategy

A comprehensive literature search was conducted in PubMed/MEDLINE, Embase.com, and Scopus from database inception without date restrictions until June 2025. These databases were selected to provide complementary coverage of biomedical, clinical, and multidisciplinary literature. Nevertheless, restricting the electronic search to these three databases may have omitted eligible studies indexed elsewhere, which is acknowledged as a limitation. The search strategy combined controlled vocabulary terms and free-text keywords related to T1DM, glycemic control, saliva, and oral microbial outcomes. Complete database-specific search strings, including field tags, Boolean operators, and the date of the final search, are provided in Supplementary Table S1 for reproducibility.

### Study selection

**Fig. 1:**
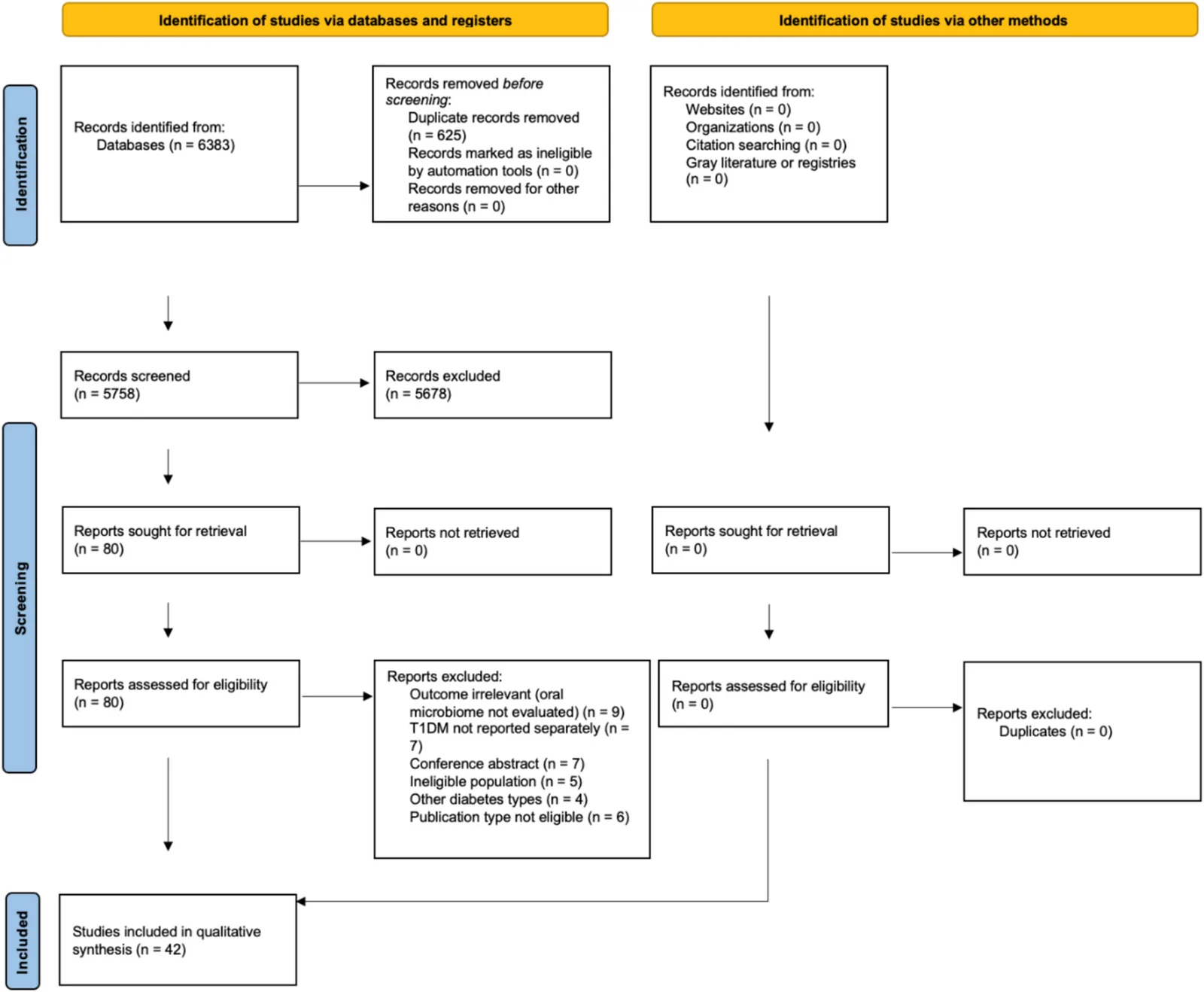
PRISMA 2020 flow diagram

All records retrieved from the electronic searches were imported into reference management software (Rayyan Online Software, Qatar Computing Research Institute), and duplicate entries were removed. Titles and abstracts were screened independently by two reviewers (AP, MD) to assess eligibility. Full-text articles were obtained for all studies deemed potentially eligible or when eligibility could not be determined from the abstract alone. Full-text screening was conducted independently by the same reviewers using the predefined eligibility criteria. Disagreements were resolved through discussion, and when necessary, by consultation with a third reviewer (EP). Reasons for exclusion at the full-text screening stage were documented. The study selection process is presented in a PRISMA flow diagram (Fig. 1).

### Data extraction

Data extraction was performed by one reviewer (AP) using a standardised data extraction form and subsequently verified by a second reviewer (MD) for accuracy and completeness. The following information was extracted from each included study: authors, publication year, study design, study population, glycemic control assessment, oral sampling site, microbiome analysis method, and the main microbiome-related findings. Comparator groups, when applicable, were also recorded. Any discrepancies identified during the verification process were resolved through discussion and consensus. When required information was missing or unclear, the corresponding authors were contacted for clarification.

### Risk of bias assessment

The methodological quality of the included studies was independently assessed by one reviewer (AP) and checked by a second reviewer (PNP) using the appropriate Joanna Briggs Institute (JBI) Critical Appraisal Checklists according to the study design. Specifically, the JBI Checklist for Analytical Cross-Sectional Studies was used for cross-sectional studies, the JBI Checklist for Cohort Studies for prospective and retrospective cohort studies, the JBI Checklist for Case-Control Studies for case-control studies, and the JBI Checklist for Quasi-Experimental Studies (Non-randomized Experimental Studies) for prospective non-randomized intervention studies. Any disagreements were resolved through discussion and, when necessary, consultation with a third reviewer.

The JBI checklists were used for domain-level critical appraisal rather than as validated numerical scoring instruments. Overall low, moderate, or high risk-of-bias judgments were assigned by reviewer consensus based on the pattern and importance of unmet or unclear domains, with particular attention to participant selection, exposure/outcome measurement, identification and management of confounding, and completeness of follow-up where applicable.

### Data synthesis

Due to anticipated and observed heterogeneity across studies, particularly regarding study design, participant characteristics, oral sampling sites, and microbiome assessment methodologies, quantitative meta-analysis was not considered appropriate. Consequently, a qualitative synthesis was conducted for all included studies. Findings were summarised descriptively, focusing on changes in the composition, diversity, or abundance of the oral microbiome associated with deregulated insulin-dependent diabetes. Where available, reported effect estimates, correlation coefficients, or comparative statistics were extracted and presented narratively or in tabular form to facilitate comparison across studies. No statistical pooling of outcomes was performed.

## Results

### Study selection

A total of 6383 records were identified through database searching. After removal of 625 duplicates, 5758 records underwent title and abstract screening, and 80 full-text articles were assessed for eligibility. Forty-two studies fulfilled the inclusion criteria and were included in the qualitative synthesis (Fig. 1).

### Characteristics of the included studies

The characteristics of the included studies are summarized in Table 1. Among the 42 included studies, 30 (71.4%) employed an analytical cross-sectional design, 6 (14.3%) were case-control studies, 4 (9.5%) were cohort (longitudinal) studies, and 2 (4.8%) were prospective non-randomized interventional studies. Sample sizes ranged from 6 to 900 participants. Most investigations were conducted in children and adolescents (*n* = 24), while the remaining studies enrolled adult populations with T1DM.

**Table 1:** Characteristics of the included studies

Authors (Year)	Study design	Population (*n*)	Glycemic control assessment	Oral sampling site	Microbiome analysis	Main findings
Sakalauskiene et al., 2014 [16]	Cross-sectional	56 adults (28 T1DM with chronic periodontitis; 28 healthy controls), 19–50 years	HbA1c; good control ≤ 7%, poor control > 7%	Supra- and subgingival plaque	DNA-based Micro-IDent Plus assay for 11 periodontal pathogens + culture for *Streptococcus intermedius*	Individuals with type 1 diabetes demonstrated a higher susceptibility to periodontal disease, with the prevalence of periodontitis rising significantly alongside poorer metabolic control.
El-Tekeya et al., 2012 [17]	Cross-sectional	100 children (50 T1DM; 50 healthy controls), 6–9 years	HbA1c: good (6–8.5%), fair (9–10%), poor (≥ 11%)	Saliva	Culture of *Mutans streptococci*, *Lactobacillus*spp. and *Candida* spp.	Metabolic control did not significantly affect microbial counts. *Mutans streptococci* was the only microbiological factor significantly associated with caries experience in diabetic children.
Merchant et al., 2016 [18]	Cross-sectional	176 adolescents (105 T1DM; 71 controls), 12–19 years	HbA1c (continuous); diabetes duration ≥ 5 years	Subgingival plaque	DNA–DNA hybridization of 41 periodontal microorganisms	Cardiovascular disease (CVD) risk factors were not found to be linked to periodontal microorganisms in youth with T1DM who maintained good oral health.
Nageeb et al., 2025 [19]	Cross-sectional	63 children (22 poorly controlled T1DM; 18 well-controlled T1DM; 23 healthy controls), 6–15 years	HbA1c: poor ≥ 7.5%; good < 7.5%	Saliva and dental plaque	Culture of *Mutans streptococci*, *Lactobacillus*spp. and *Candida* spp. (species identification and colony counts)	Despite the absence of a significant relationship between diabetes and risk of caries, the oral microbiologic profile was distinct between healthy and diabetic pediatric cohorts - significantly modulated by glycemic control.
Sastrowijoto et al., 1990 [20]	Cross-sectional	6 adults with IDDM and poor metabolic control	HbA1c measured every 4 months during intensive insulin therapy	Healthy and diseased subgingival periodontal pockets	Culture-based identification of subgingival microbiota (including *Actinobacillus actinomycetemcomitans*, black-pigmented *Bacteroides*, *Capnocytophaga*, *Staphylococcus aureus*, *Candida albicans*)	Improvement in the periodontal condition of IDDM patients may be contingent upon the implementation of local oral hygiene.
Ferizi et al., 2022 [21]	Case-control	80 children with T1DM (34 good control; 46 poor control), 10–15 years	HbA1c < 7.5% vs. > 7.5%	Stimulated saliva	CRT Bacteria test for *Streptococcus mutans*and *Lactobacillus*	Pediatric diabetic patients present with poorer oral hygiene and face an elevated risk for both dental caries and periodontal diseases, especially when metabolic control is inadequate.
Twetman et al., 2002 [22]	Prospective cohort (3-year follow-up)	64 children/adolescents with newly diagnosed T1DM, 8–15 years	Mean HbA1c during follow-up (good vs. less good metabolic control)	Whole saliva	Cultures of salivary *Mutans streptococci* and *Lactobacillus*	In children and adolescents with type 1 diabetes mellitus, both the degree of metabolic control and traditional caries risk markers represent critical determinants of caries development.
Swanljung et al., 1992 [23]	Case-control	85 adolescents with well-controlled IDDM and 85 healthy controls	HbA1c (metabolically well-controlled diabetes)	Stimulated saliva	Culture-based quantification of *Mutans streptococci* and *Lactobacillus*; salivary glucose	Patients with well-controlled IDDM exhibit salivary and caries data comparable to those of healthy controls.
Moskovitz et al., 2021 [24]	Cross-sectional	37 children with T1DM and 29 age- and sex-matched healthy controls (5–15 years)	T1DM diagnosis (glycemic control not used for group stratification)	Unstimulated whole saliva	16 S rRNA sequencing	Salivary microbiome analysis revealed distinct taxa in T1D children, including several genera associated with the T1D gut microbiome.
Chakraborty et al., 2021 [25]	Cross-sectional	60 adolescents (20 T1DM + periodontal disease, 20 T1DM without periodontal disease, 20 periodontal disease only)	Mean HbA1c over the previous year	Gingival plaque	Culture-based microbial identification (Vitek 2) and biofilm assay	Poor glycemic control in pediatric T1DM drives increased biofilm formation and microbial diversity, a trend most pronounced in patients with periodontal disease.
Yuan et al., 2022 [26]	Cross-sectional	46 new-onset T1DM, 10 chronic T1DM receiving insulin, and 47 healthy children	Acute vs. chronic T1DM; correlations with HbA1c and fasting blood glucose	Saliva	16 S rRNA sequencing	The acute phase of T1D exhibited oral microbiota dysbiosis, which was partially mitigated by glycemic control.
de Groot et al., 2017 [27]	Observational case-control	53 adults with longstanding T1DM and 50 healthy controls	HbA1c; excluded poorly controlled patients (HbA1c > 10%)	Oral swabs (upper labial sulcus) and feces	16 S rRNA sequencing	T1D is characterized by altered intestinal butyrate-producer abundance and distinct plasma acetate and propionate levels.
Babatzia et al., 2020 [28]	Cross-sectional	74 children with T1DM (good vs. poor glycemic control) and 70 healthy controls	HbA1c (< 7.5% vs. ≥ 7.5%)	Stimulated saliva	Real-time PCR for *Streptococcus mutans* and *Candida albicans*	Youth with T1DM exhibit poorer oral hygiene and higher disease risk, particularly under poor metabolic control, though systemic, extra-oral factors also drive disease progression.
Pachoński et al., 2021 [29]	Cross-sectional	50 children with T1DM (25 well-controlled, 25 poorly controlled) and 25 healthy controls	HbA1c (≤ 7.5% vs. > 7.5%)	Floor of the mouth and tongue dorsum swabs	Conventional microbiological culture	The oral microbiome of children with type 1 diabetes exhibits significant quantitative and qualitative differences compared to healthy controls.
Jensen et al., 2021 [30]	Cross-sectional	77 children/adolescents with T1DM	Continuous HbA1c assessment	Gingival plaque and buccal mucosa	16 S rRNA sequencing	Suboptimal glycemic control in children with type 1 diabetes was continuously correlated with an increase in early indicators of periodontal disease.
Akpata et al., 2012 [31]	Cross-sectional	53 children with T1DM (14 controlled, 39 uncontrolled) and 53 healthy controls, 12–15 years	HbA1c (< 8% vs. > 8%)	Saliva	Dentocult kits for *Streptococcus mutans*, *Lactobacillus*, and yeasts	Low resting salivary flow rates significantly drove the heightened caries experience observed in diabetic children compared to healthy controls.
López del Valle & Ocasio-López, 2011 [32]	Case-control	25 children with T1DM and 25 healthy controls, 6–12 years	HbA1c (analyzed as continuous variable)	Saliva	Culture for *Streptococcus mutans* and *Lactobacillus*	Diabetic children exhibited higher plaque indices and bleeding on probing, with the latter significantly associated with elevated glycosylated hemoglobin levels.
Twetman et al., 1992 [33]	Prospective cohort (1-year follow-up)	70 children with T1DM, 7–18 years	HbA1c categorized as well-, moderately-, or poorly-controlled	Stimulated saliva	Dentocult for *Streptococcus mutans* and *Lactobacillus*	Poorly controlled diabetes was associated with significantly higher salivary *S. mutans* levels. After placement of fluoride-releasing restorations, *S. mutans* counts decreased and no new carious lesions developed during the first year.
Siudikiene et al., 2006 [34]	Cross-sectional	68 children with T1DM and 68 matched healthy controls, 10–15 years	HbA1c (< 9% vs. ≥ 9%)	Saliva	Dentocult for *Streptococcus mutans*, *Lactobacillus*, yeasts; salivary flow and buffering capacity	While diabetic metabolic control did not affect saliva, caries susceptibility was driven by plaque accumulation, older age, and reduced unstimulated salivary flow.
Bolgül et al., 2004 [35]	Prospective cohort (1-year follow-up)	70 children with insulin-dependent diabetes mellitus (IDDM), 4–15 years	Mean HbA1c from the previous 6 months; well-controlled:<10%, moderately controlled: 10.0–12.9%, poorly controlled:≥13%	Paraffin-stimulated whole saliva	CRT Bacteria test (Vivadent) for *Streptococcus mutans* and *Lactobacillus*; salivary pH	Salivary MS levels remained unaffected by glycemic control, whereas lactobacilli populations were reduced regardless of metabolic status.
Gusberti et al., 1983 [36]	Cross-sectional	77 children with type 1 diabetes, 6–15 years	Fasting glucose and HbA1c	Marginal/interproximal plaque	Total cultivable flora, *Capnocytophaga*, *Actinomyces naeslundii*, black-pigmented *Bacteroides*, *S. mutans*, *S. sanguis*, *Veillonella*, *Fusobacterium*	While prepubertal gingivitis tracks with glycemic control, puberty triggers independent gingival inflammation and specific shifts in marginal plaque microbiota.
Sbordone et al., 1995 [37]	Cross-sectional (matched sibling controls)	16 children with T1DM and 16 healthy siblings	Mean HbA1c 8.76%	Subgingival plaque	*Microbial cultural analysis Porphyromonas gingivalis*, *Prevotella intermedia*, *Actinobacillus actinomycetemcomitans*, *Capnocytophaga* spp.	While overall microflora was similar, a specific juvenile diabetic cluster with high P. gingivalis exhibited significantly greater periodontal attachment loss and probing depth.
Schara et al., 2013 [38]	Cross-sectional observational study	21 adults (40–50 years) with type 1 diabetes and periodontitis	HbA1c (%) measured from blood samples (mean 7.2 ± 0.7%)	Subgingival plaque from the two deepest periodontal pockets	Multiplex PCR (micro-IDent) for *Aggregatibacter actinomycetemcomitans*,* Porphyromonas gingivalis*,* Prevotella intermedia*,* Tannerella forsythia*, and *Treponema denticola*	*T. forsythia and T. denticola are most frequently found in T1D subgingival plaque*,* correlating directly with poorer metabolic control.*
Sbordone et al., 1998 [39]	3-year longitudinal cohort	16 children/adolescents with T1DM and 16 healthy sibling controls	HbA1c monitored every 3 months throughout the study	Subgingival plaque from one constant site and one deepest periodontal site	Anaerobic culture for *Porphyromonas gingivalis*,* Prevotella intermedia*,* Actinobacillus (Aggregatibacter) actinomycetemcomitans*,and *Capnocytophaga*spp.	Patients with diabetes and their non-diabetic siblings showed no significant differences in periodontal parameters over the 3-year longitudinal study
Sastrowijoto et al., 1989 [40]	Cross-sectional	22 adults with T1DM (12 near-normal metabolic control; 10 poor metabolic control)	HbA1c: near-normal < 7.7%; poor > 9.9%	One healthy (≤ 3 mm) and one diseased (> 4 mm) periodontal pocket per patient	Anaerobic culture for *Actinobacillus (Aggregatibacter) actinomycetemcomitans*, black-pigmented *Bacteroides* (*P. gingivalis*, *P. intermedia*), and *Capnocytophaga* spp.	Independent of diabetic metabolic control, periodontal pathogenesis in T1D is driven by A. actinomycetemcomitans and black-pigmented Bacteroides rather than previously overestimated Capnocytophaga species.
Seppää & Ainamo 1996 [41]	Cross-sectional study	47 adults with long-term T1DM (26 poorly controlled, 21 controlled)	Long-term medical records, blood glucose and HbA1/HbA1c levels	106 subgingival sites (55 healthy sulci and 51 diseased periodontal pockets)	Dark-field microscopy evaluating bacterial morphotypes (spirochetes, motile rods, cocci, rods, filaments, fusiforms)	Poorly controlled insulin-dependent diabetes patients exhibited worse periodontal parameters (plaque, bleeding, attachment, and bone loss) and significantly higher subgingival spirochetes and motile rods, but fewer coccoid cells, than controlled subjects.
Carelli et al., 2023 [13]	Cross-sectional	89 children and adolescents with T1DM (mean age 12.6 ± 2.2 years)	HbA1c, glucose management indicator (GMI), time in range (TIR), time above range (TAR), coefficient of variation (CGM-derived metrics)	Saliva	Culture and multiplex PCR for cariogenic and periodontal bacteria (*S. mutans*,* Veillonella spp.*,* Lactobacillus spp.*,* Actinomyces spp.*,* A. actinomycetemcomitans*,* P. intermedia*,* P. gingivalis*,* T. denticola*,* T. forsythia*)	Good oral hygiene habits negatively correlate with red complex pathogens (T. forsythia, T. denticola, P. gingivalis), highlighting the need for glycemic control and hygiene in pediatric T1D.
Maris et al., 2024 [42]	Cross-sectional	42 adults with type 1 diabetes undergoing oral rehabilitation	HbA1c (range 5.9–9.6%)	Subgingival plaque from the two deepest periodontal pockets	Quantitative real-time PCR (qPCR) for *Aggregatibacter actinomycetemcomitans*,* Porphyromonas gingivalis*,* Prevotella intermedia*,* Tannerella forsythia*, and *Treponema denticola*	In type 1 diabetes, subgingival pathogen presence is driven by both increased pocket probing depth and poor metabolic control.
Karjalainen et al., 1997 [43]	Cross-sectional	80 children and adolescents with T1DM (mean age 14.5 years)	HbA1: well-controlled < 10%, moderately controlled 10–12.9%, poorly controlled > 13%	Saliva	Culture of yeasts, Streptococcus mutans, and Lactobacillus; salivary flow, pH, and buffering capacity	Poor glycemic control significantly alters saliva by increasing yeast prevalence, whereas clinical salivary flow, pH, and bacterial counts show no direct association.
Edblad et al., 2001 [44]	Cross-sectional	149 patients with insulin-dependent diabetes mellitus	HbA1c (< 8.5% vs. ≥ 8.5%)	Saliva	Quantification of *Streptococcus mutans* and *Lactobacillus*; salivary factors (flow rate, pH, buffering capacity)	No significant differences were observed in salivary status, including flow rate, buffering capacity, and levels of Streptococcus mutans or Lactobacilli.
Canepari et al., 1994 [45]	Cross-sectional	Young patients with T1DM with varying glyco-metabolic control and healthy controls	Glyco-metabolic control (HbA1c/glycemic balance; well-controlled vs. poorly controlled)	Saliva	Culture of *Streptococcus mutans* and *Lactobacillus*; salivary flow rate, pH, buffering capacity, and glucose concentration	Insulin-dependent diabetes causes salivary and subgingival dysbiosis, enriching periopathogens like Porphyromonas and Prevotella, though effective glycemic control partially restores healthy microbial diversity.
Olczak-Kowalczyk et al., 2015 [46]	Cross-sectional	35 children/adolescents with T1DM , 32 with nephrotic syndrome, and 29 healthy controls	Blood glucose; HbA1c (compensated < 8% vs. uncompensated > 8%)	Oral mucosa	Oral swabs for Candida culture; species identification; assessment of Candidaenzymatic activity (valine arylamidase, α-glucosidase, N-acetyl-β-glucosaminidase)	Poor glycemic control correlates with severe gingivitis in diabetes, where Candida colonization potentially exacerbates plaque-induced periodontal inflammation.
Kumar et al., 2005 [47]	Cross-sectional	49 patients with T1DM, 54 patients with type 2 diabetes, and 100 healthy controls	Glycemic control (anti-diabetic therapy and HbA1c/metabolic control)	Oral cavity (oral swab)	Oral Candida culture; colony-forming unit (CFU) counts	Candida colonization rate and density (CFU) are significantly higher in the oral cavity of type 1 and type 2 diabetics compared to healthy controls, showing no direct relation to glycemic control.
Aly et al., 1992 [48]	Cross-sectional	Individuals with insulin-dependent diabetes (IDDM), non-insulin-dependent diabetes (NIDDM), and healthy subjects (total diabetic cohort: 439)	HbA1, plasma glucose levels, glycosuria	Oral cavity (oral rinse) and palate (palatal swab)	Culture of oral yeasts (*Candida*spp.)	Oral yeast carriage in IDDM is driven by age and glycemic control, while NIDDM carriage is influenced by glycemic control, salivary ABH non-secretion, and denture wear.
Willis et al., 1999 [49]	Cross-sectional	414 insulin-treated diabetes mellitus patients	Glycemic control (HbA1c/HbA1 or clinical glycemic status)	Oral cavity (oral rinse)	Oral rinse culture for *Candida* spp.; species identification; candidal load quantification	Oral Candida carriage in diabetics is dominated by C. albicans and C. dubliniensis, unaffected by glycemic control.
Buzininet al., 2014 [50]	Prospective controlled clinical study	20 patients with T1DM and chronic periodontitis; 21 non-diabetic patients with chronic periodontitis	HbA1c (baseline and 3 months after nonsurgical periodontal therapy)	Subgingival plaque	Quantification of periodontal pathogens from plaque samples *(verify method from full text; likely PCR or culture)*	Nonsurgical periodontal therapy in type 1 diabetics significantly reduces periodontal pathogens without differing in clinical periodontal parameters compared to non-diabetic controls.
Guggenheimer et al., 2000 [51]	Cross-sectional	405 patients with insulin-dependent diabetes mellitus (IDDM) and 268 non-diabetic controls	Glycosylated hemoglobin (HbA1c)	Midline posterior dorsal tongue	Cytological smear with quantitative assessment of *Candida albicans*pseudohyphae; clinical assessment of oral candidiasis	IDDM patients show a higher prevalence of oral Candida pseudohyphae, which is significantly associated with poor glycemic control, smoking, and denture use.
Manfredi et al., 2002 [52]	Cross-sectional	56 patients with T1DM and 81 patients with type 2 diabetes	HbA1c (glycosylated hemoglobin)	Oral rinse	Oral rinse culture; yeast quantification (CFU/mL); phenotypic and molecular identification of *Candida* spp.; genotyping of *C. albicans*	Local oral factors, particularly denture wear, influence oral Candida species and load in diabetics more than diabetic status itself.
Hropot Plesko et al., 2021 [53]	Prospective interventional clinical study	11 patients with T1DM, 11 patients with T2DM, and 11 non-diabetic patients with periodontitis	HbA1c measured at baseline and 3 months after full-mouth disinfection (FMD)	Subgingival plaque (two deepest periodontal pockets)	PCR detection of *Aggregatibacter actinomycetemcomitans*(*Aa*), *Porphyromonas gingivalis* (*Pg*), *Prevotella intermedia*(*Pi*), *Tannerella forsythia* (*Tf*), and *Treponema denticola*(*Td*)	Full-mouth disinfection (FMD) significantly improves periodontal parameters (BOP, PPD, CAL) and glycemic control in type 1 diabetics, yet triggers a significant increase in the oral pathogen Aggregatibacter actinomycetemcomitans (Aa).
Duggal et al., 2021 [54]	cross-sectional	470 diabetic patients (including Type 1 and Type 2) and 430 non-diabetic controls	HbA1c; diabetic control categorized using HbA1c > 7%	Oral swab (oral cavity)	Culture and species identification of *Candida* spp.	Type 1 diabetics exhibit a high oral Candida prevalence (22.6%, predominantly C. albicans), which is closely associated with poor oral hygiene and elevated glycemic levels (HbA1c > 7%).
Shenoy et al., 2014 [55]	Case-control	30 patients T1DM, 30 patients with T2DM, and 30 healthy controls	Fasting blood glucose (FBG) and HbA1c	Saliva	Culture on Sabouraud dextrose agar (SDA) and chromogenic agar; quantification of *Candida*colony-forming units (CFU/mL)	Oral Candida load is significantly higher in type 1 and 2 diabetics, directly associating with poor glycemic control (HbA1c%).
Al-Attas & Amro, 2010 [56]	Case-control	49 patients with T1DM, 101 patients with T2DM, and 50 healthy controls	Glycemic control (HbA1c/clinical diabetic control; verify from full text)	Saliva (oral rinse)	Oral rinse culture; *Candida* species identification; antifungal susceptibility testing (Candifast)	Diabetics exhibit higher oral Candida carriage rates and increased azole antifungal resistance compared to controls, with colonization patterns driven by diabetes type, salivary pH, and glycemic control.

Healthy age- and sex-matched individuals served as the most common comparator. Several studies additionally compared participants according to glycemic control, disease duration, or periodontal status, whereas a small number also included T2DM cohorts or other comparison groups (e.g., sibling controls or nephrotic syndrome); however, only data from participants with T1DM were considered in the present review.

**Fig. 2:**
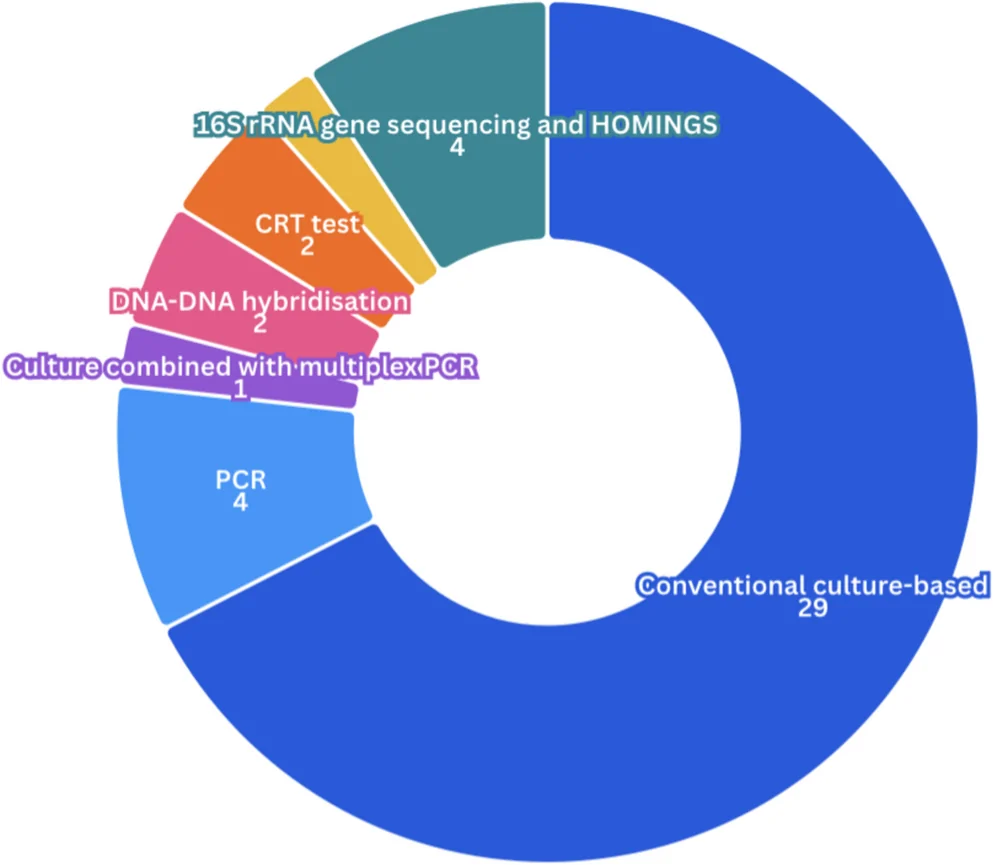
Microbiological methodologies varied across the included studies

Microbiological methodologies varied substantially across the included studies. Conventional culture-based techniques were the most frequently employed analytical approach (*n* = 29), primarily for the detection of cariogenic microorganisms, *Candida* spp., and selected periodontal pathogens. Molecular techniques were applied in seven studies, including conventional PCR (*n* = 4), culture combined with multiplex PCR (*n* = 1), and DNA–DNA hybridisation (*n* = 2). Two studies used the CRT test, one employed dark-field microscopy, whereas only four studies applied culture-independent microbiome profiling approaches, including 16 S rRNA gene sequencing and HOMINGS (Moskovitz 2021, de Groot 2017, Jensen 2021, and Yuan 2022), to characterise the overall oral microbial community (Fig. 2).

Detailed quantitative findings regarding associations between glycemic control and oral microbial or clinical outcomes, including reported HbA1c values, correlation coefficients, microbial prevalence or abundance measures, effect estimates, and measures of statistical significance, are presented in Supplementary Table S1.

Glycemic control was assessed predominantly using HbA1c, but definitions varied markedly. Among studies that dichotomised or categorised control, reported thresholds included approximately 7.0%, 7.5%, 8.0%, 9.0%, 10.0%, and, in older studies, thresholds as high as 13% for poor control; other studies analysed HbA1c continuously or used longitudinal averages. The study-specific definitions are presented in Table 1. This heterogeneity limits direct comparison between studies and may contribute to inconsistent microbial findings.

**Fig. 3:**
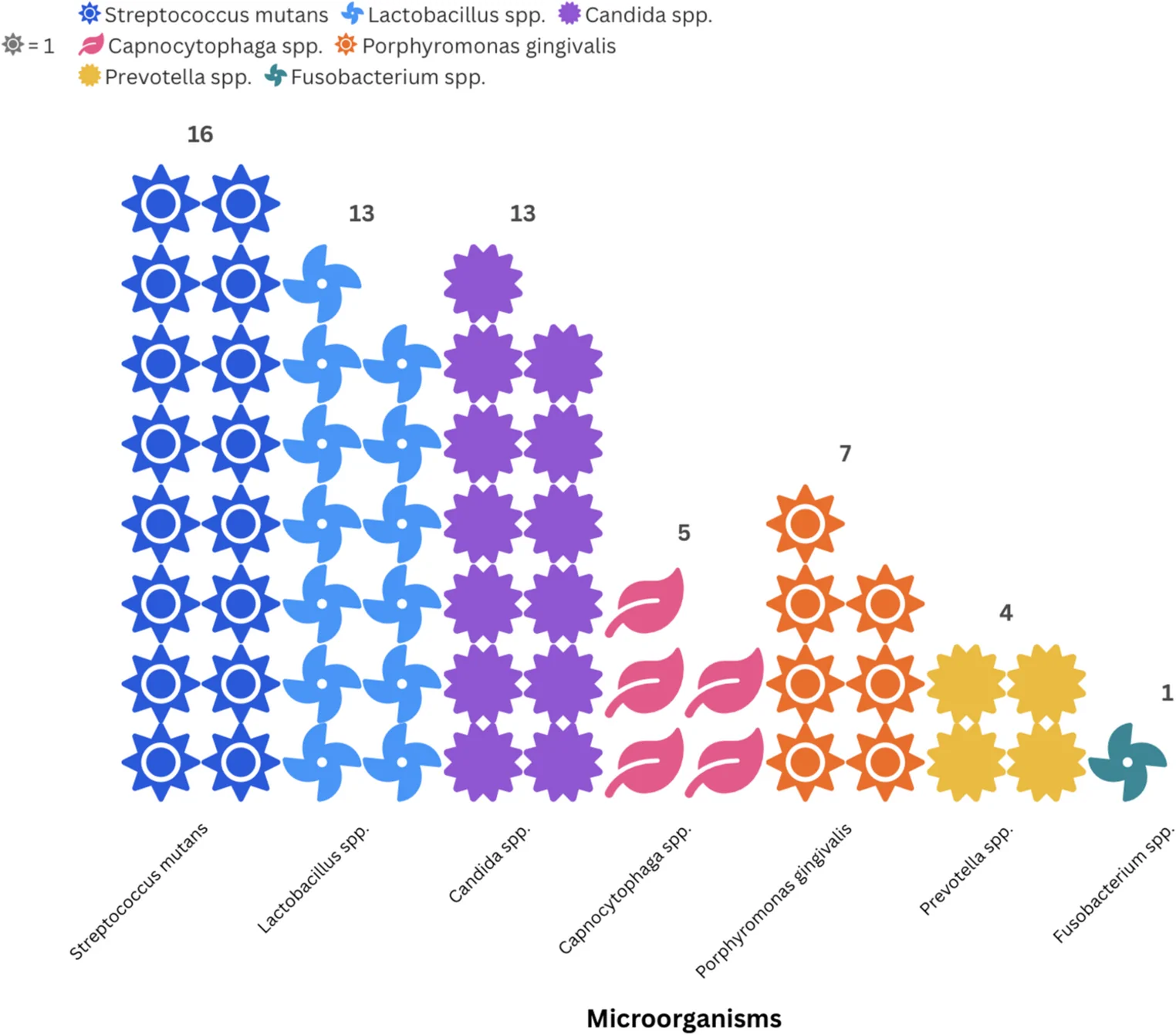
Frequency of the microorganisms investigated in the included studies. Each icon represents one study, and the numbers above each column indicate the total number of studies evaluating the respective microorganism

Saliva and subgingival plaque were the most frequently sampled oral niches, with saliva collected in 18 studies and periodontal samples analyzed in 16 studies. Other sampling sites included supragingival plaque, oral mucosa, tongue dorsum, palate, oral rinse, and healthy or diseased periodontal pockets. The microorganisms most frequently investigated were Streptococcus mutans (*n* = 16), Lactobacillus spp. (*n* = 13), Candida spp. (*n* = 13), Capnocytophaga spp. (*n* = 5), Porphyromonas gingivalis (*n* = 7), Prevotella spp. (*n* = 4), and Fusobacterium spp. (*n* = 1) (Fig. 3).

Clinical oral disease status also varies substantially. Included populations ranged from participants with clinically healthy periodontia or good oral health to cohorts selected for periodontitis, gingivitis, caries experience, or mixed oral-health status. Several pediatric studies reported plaque accumulation, gingival bleeding, caries experience, salivary-flow measures, or periodontal indices alongside microbial outcomes. Because periodontal inflammation and caries activity can themselves shape local microbial communities, microbial differences cannot be attributed to glycemic control without considering the underlying oral disease phenotype. Study-level oral disease characteristics should therefore be interpreted together with the microbial findings in Table 1.

### Periodontal microbiota

Sixteen studies specifically investigated periodontal microorganisms. The most frequently evaluated taxa were Porphyromonas gingivalis, Tannerella forsythia, Treponema denticola, Aggregatibacter actinomycetemcomitans, and Prevotella intermedia. Several studies reported higher prevalence or abundance of one or more targeted periodontal pathogens in participants with poorer glycemic control, whereas other investigations observed no significant microbiological differences despite worse clinical periodontal parameters. Thus, the targeted periodontal literature does not demonstrate a uniform community-wide dysbiosis signal. Two prospective interventional studies reported improvements in periodontal clinical measures and HbA1c after periodontal therapy, while microbiological changes were limited; these findings do not establish that any glycemic improvement was mediated by microbiome change.

### Salivary and cariogenic microbiota

Eighteen studies evaluated saliva, including five that specifically analyzed stimulated saliva. Most focused on Streptococcus mutans, Lactobacillus spp., and salivary characteristics, whereas three studies applied sequencing-based approaches to characterize the salivary microbiome. Several investigations reported higher S. mutans counts, reduced salivary flow, and increased caries experience among individuals with poorer glycemic control. However, associations between HbA1c and cariogenic bacterial counts were inconsistent across studies. Sequencing-based studies consistently identified differences in salivary microbial composition between individuals with T1DM and healthy controls.

### Oral *Candida*

Thirteen studies investigated oral Candida colonization. Most reported higher oral yeast carriage among individuals with T1DM than healthy controls, with Candida albicans representing the predominant species. Several studies demonstrated positive associations between HbA1c or fasting blood glucose and oral candidal colonization, whereas others found no significant relationship between glycemic control and candidal carriage. Non-albicans Candida species, including C. dubliniensis, C. glabrata, and C. tropicalis, were identified less frequently.

### Risk of bias assessment

Overall, the methodological quality of the included studies was considered satisfactory. Of the 42 included studies, 17 (40.5%) were judged to have a low risk of bias, while 25 (59.5%) were assessed as having a moderate risk of bias. No study was considered to be at high risk of bias. The most common methodological limitations included inadequate control of potential confounding factors, limited reporting of strategies to address confounding, relatively small sample sizes, and the absence of blinded outcome assessment in prospective non-randomized studies. Detailed results of the methodological quality assessment for each study are presented in Table 2.

**Table 2:** Methodological quality assessment for each study

Authors (Year)	Study design	Tool	Risk of bias
Sakalauskiene et al., 2014 [16]	Cross-sectional	JBI Critical Appraisal Checklist for Cross-Sectional Studies	Moderate
El-Tekeya et al., 2012 [17]	Cross-sectional	JBI Critical Appraisal Checklist for Cross-Sectional Studies	Low
Merchant et al., 2016 [18]	Cross-sectional	JBI Critical Appraisal Checklist for Cross-Sectional Studies	Low
Nageeb et al., 2025 [19]	Cross-sectional	JBI Critical Appraisal Checklist for Cross-Sectional Studies	Moderate
Sastrowijoto et al., 1990 [20]	Cross-sectional	JBI Critical Appraisal Checklist for Cross-Sectional Studies	Moderate
Ferizi et al., 2022 [21]	Case-control	JBI Critical Appraisal Checklist for Case-Control Studies	Low
Twetman et al., 2002 [22]	Prospective cohort (3-year follow-up)	JBI Critical Appraisal Checklist for Cohort Studies	Low
Swanljung et al., 1992 [23]	Case-control	JBI Critical Appraisal Checklist for Case-Control Studies	Moderate
Moskovitz et al., 2021 [24]	Cross-sectional	JBI Critical Appraisal Checklist for Cross-Sectional Studies	Low
Chakraborty et al., 2021 [25]	Cross-sectional	JBI Critical Appraisal Checklist for Cross-Sectional Studies	Moderate
Yuan et al., 2022 [26]	Cross-sectional	JBI Critical Appraisal Checklist for Cross-Sectional Studies	Moderate
de Groot et al., 2017 [27]	Observational case-control	JBI Critical Appraisal Checklist for Case-Control Studies	Low
Babatzia et al., 2020 [28]	Cross-sectional	JBI Critical Appraisal Checklist for Cross-Sectional Studies	Low
Pachoński et al., 2021 [29]	Cross-sectional	JBI Critical Appraisal Checklist for Cross-Sectional Studies	Moderate
Jensen et al., 2021 [30]	Cross-sectional	JBI Critical Appraisal Checklist for Cross-Sectional Studies	Low
Akpata et al., 2012 [31]	Cross-sectional	JBI Critical Appraisal Checklist for Cross-Sectional Studies	Low
López del Valle & Ocasio-López, 2011 [32]	Case-control	JBI Critical Appraisal Checklist for Case-Control Studies	Moderate
Twetman et al., 1992 [33]	Prospective cohort (1-year follow-up)	JBI Critical Appraisal Checklist for Cohort Studies	Moderate
Siudikiene et al., 2006 [34]	Cross-sectional	JBI Critical Appraisal Checklist for Cross-Sectional Studies	Low
Bolgül et al., 2004 [35]	Prospective cohort (1-year follow-up)	JBI Critical Appraisal Checklist for Cohort Studies	Moderate
Gusberti et al., 1983 [36]	Cross-sectional	JBI Critical Appraisal Checklist for Cross-Sectional Studies	Moderate
Sbordone et al., 1995 [37]	Cross-sectional (matched sibling controls)	JBI Critical Appraisal Checklist for Cross-Sectional Studies	Low
Schara et al., 2013 [38]	Cross-sectional observational study	JBI Critical Appraisal Checklist for Cross-Sectional Studies	Moderate
Sbordone et al., 1998 [39]	3-year longitudinal cohort	JBI Critical Appraisal Checklist for Cohort Studies	Moderate
Sastrowijoto et al., 1989 [40]	Cross-sectional comparative study	JBI Critical Appraisal Checklist for Cross-Sectional Studies	Moderate
Seppää & Ainamo, 1996 [41]	Cross-sectional study	JBI Critical Appraisal Checklist for Cross-Sectional Studies	Moderate
Carelli et al., 2023 [13]	Cross-sectional	JBI Critical Appraisal Checklist for Cross-Sectional Studies	Low
Maris et al., 2024 [42]	Cross-sectional	JBI Critical Appraisal Checklist for Cross-Sectional Studies	Moderate
Karjalainen et al., 1997 [43]	Cross-sectional	JBI Critical Appraisal Checklist for Cross-Sectional Studies	Moderate
Edblad et al., 2001 [44]	Cross-sectional	JBI Critical Appraisal Checklist for Cross-Sectional Studies	Moderate
Canepari et al., 1994 [45]	Cross-sectional	JBI Critical Appraisal Checklist for Cross-Sectional Studies	Moderate
Olczak-Kowalczyk et al., 2015 [46]	Cross-sectional	JBI Critical Appraisal Checklist for Cross-Sectional Studies	Low
Kumar et al., 2005 [47]	Cross-sectional	JBI Critical Appraisal Checklist for Cross-Sectional Studies	Moderate
Aly et al., 1992 [48]	Cross-sectional	JBI Critical Appraisal Checklist for Cross-Sectional Studies	Low
Willis et al., 1999 [49]	Cross-sectional	JBI Critical Appraisal Checklist for Cross-Sectional Studies	Moderate
Buzinin et al., 2014 [50]	Prospective controlled clinical study	JBI Quasi-Experimental Checklist	Moderate
Guggenheimer et al., 2000 [51]	Cross-sectional	JBI Critical Appraisal Checklist for Cross-Sectional Studies	Low
Manfredi et al., 2002 [52]	Cross-sectional	JBI Critical Appraisal Checklist for Cross-Sectional Studies	Moderate
Hropot Pleško et al., 2021 [53]	Prospective interventional clinical study	JBI Quasi-Experimental Checklist	Moderate
Duggal et al., 2021 [54]	cross-sectional	JBI Critical Appraisal Checklist for Cross-Sectional Studies	Moderate
Shenoy et al., 2014 [55]	Case-control	JBI Critical Appraisal Checklist for Case-Control Studies	Low
Al-Attas & Amro, 2010 [56]	Case-control	JBI Critical Appraisal Checklist for Case-Control Studies	Moderate

## Discussion

This systematic review synthesized evidence on associations between glycemic control and oral microbial findings in individuals with T1DM. The evidence base is dominated by cross-sectional studies and targeted culture or molecular assays. Accordingly, the most defensible conclusion is that poorer glycemic control is associated in some studies with altered levels or detection of selected periodontal taxa and with oral Candida carriage, while findings for cariogenic microorganisms are heterogeneous. Only four studies used comprehensive culture-independent community profiling; these provide preliminary evidence of differences in community composition and/or diversity but are insufficient to establish a consistent direction of whole-community dysbiosis or a causal effect of hyperglycemia. The strongest evidence in this review concerned periodontal microorganisms. Most studies reported higher prevalence or abundance of red-complex bacteria, particularly Porphyromonas gingivalis, Tannerella forsythia, and Treponema denticola, among individuals with poorer metabolic control. These findings align with previous reviews showing that T1DM is associated with increased periodontal destruction and greater susceptibility to periodontitis, particularly in patients with elevated HbA1c levels [8, 57]. Hyperglycemia may modify the subgingival environment by increasing advanced glycation end-products, oxidative stress, altered neutrophil function, and exaggerated inflammatory responses, thereby favouring colonisation by periodontal pathogens rather than directly initiating infection [8, 57–59].Clinical periodontal severity and pathogen detection may diverge because periodontal disease reflects host inflammatory response, cumulative tissue destruction, plaque ecology, sampling depth/site, treatment history, and other exposures in addition to the abundance of individual organisms. Age is also an important source of heterogeneity: more than half of the studies included children or adolescents, in whom periodontal pathogen prevalence and established periodontitis differ from adult populations. The present evidence therefore should not be interpreted as demonstrating increased periodontal pathogens across all ages or all T1DM populations; associations appear context-dependent and are particularly difficult to separate from periodontal status and age.

In contrast, the association between glycemic regulation and cariogenic microorganisms was less consistent. While several studies demonstrated increased Streptococcus mutans counts or higher caries prevalence in individuals with poorer metabolic control, others found no differences in cariogenic bacterial levels despite greater caries experience. Because many included caries-focused studies sampled saliva rather than supragingival plaque, differences in sampling site may partly explain inconsistent findings for Streptococcus mutans and Lactobacillus spp. Caries is also multifactorial; salivary flow and buffering capacity, diet, oral hygiene, fluoride exposure, plaque accumulation, age, and host factors can modify both disease risk and microbial measurements [60]. Poor glycemic control should therefore be considered one potential correlate within this broader clinical and ecological context rather than an independent determinant of cariogenic microbial change.

Salivary biomarker studies further support this concept, suggesting that host immune responses also contribute to caries development alongside microbial dysbiosis [61]. Similarly, recent salivary proteomic evidence indicates that alterations in host-derived salivary proteins may further increase caries susceptibility in individuals with poor glycemic control [62]. Salivary flow rate, buffering capacity, dietary habits, oral hygiene practices, fluoride exposure, and plaque accumulation are well-established determinants of caries risk and were also frequently identified as important modifiers across the included studies, suggesting that poor glycemic control may amplify, rather than independently determine, cariogenic microbial changes [63, 64].The evidence regarding oral fungal colonization was comparatively more consistent. Most studies reported increased oral Candida carriage among individuals with T1DM, with Candida albicans representing the predominant species [52, 65]. Several investigations further demonstrated positive associations between HbA1c levels and candidal colonization or colony-forming units, although this relationship was not universal [66]. Hyperglycemia may promote fungal proliferation through increased salivary glucose concentrations, impaired neutrophil function, reduced salivary antimicrobial activity, and xerostomia, thereby creating a favorable environment for opportunistic fungal colonization [67]. Nevertheless, local factors such as denture use, smoking, antimicrobial therapy, and salivary pH were also identified as important determinants of candidal carriage, emphasizing the multifactorial nature of oral fungal dysbiosis [68–70].

One of the most notable observations from the present review is the limited application of comprehensive microbiome profiling techniques. Despite the inclusion of 42 studies, only a small proportion (*n* = 4) employed next-generation sequencing approaches such as 16 S rRNA gene sequencing or HOMINGS. Most investigations relied on culture-based methods or targeted molecular assays directed toward predefined microorganisms, which detect only cultivable or selected taxa and may therefore underestimate the complexity of the oral microbial ecosystem. Consequently, much of the available evidence reflects changes in selected pathogens rather than alterations of the oral microbial ecosystem as a whole. Nevertheless, sequencing studies consistently demonstrated reduced microbial diversity and distinct community structures in individuals with T1DM, highlighting that community-level alterations may provide more comprehensive insights than individual pathogens alone. Future investigations should therefore prioritize culture-independent technologies capable of characterizing the entire oral microbiome and move beyond taxonomic characterization toward functional profiling of microbial communities and their metabolic pathways.

The heterogeneity observed across studies should be interpreted in light of several methodological differences. Considerable variability existed in participant age, diabetes duration, definitions of metabolic control, oral sampling sites, microbiological methodologies, and outcome reporting. HbA1c thresholds defining adequate metabolic control were not consistent, while oral samples included saliva, supragingival plaque, subgingival plaque, mucosal swabs, tongue swabs, and oral rinse specimens. Furthermore, culture-based methods predominated, whereas only a minority of studies employed high-throughput sequencing technologies. These methodological differences likely contributed substantially to the variability in reported microbial associations and precluded direct quantitative comparison across studies.

From a clinical perspective, the findings emphasize the importance of maintaining optimal metabolic control not only for systemic health but also for preserving oral microbial homeostasis. The consistent associations between poor glycemic control, periodontal dysbiosis, and increased *Candida* colonization further support the integration of regular dental examinations, routine periodontal surveillance, and preventive oral healthcare from childhood into the multidisciplinary management of individuals with T1DM. Recent evidence further supports these findings, showing that children and young adults with T1DM have a higher burden of oral diseases and a distinct inflammatory profile regardless of metabolic status [71]. Early identification of oral microbial alterations may contribute to improved oral health outcomes and potentially reduce the burden of diabetes-related oral complications, while oral microbiome monitoring may represent a promising future biomarker of oral disease risk.

The present review has several strengths. To the authors’ knowledge, it represents one of the first systematic reviews specifically focusing on the relationship between glycemic regulation and the oral microbiome in T1DM. The review considered a broad range of microbial niches, analytical methods, and microbial taxa, providing a comprehensive overview of the available evidence. Nevertheless, several limitations should be acknowledged. Most included studies were cross-sectional, precluding causal inference; sample sizes were often small; definitions of glycemic control and oral disease varied; confounder adjustment was inconsistent; and most microbial assessments were culture-based or targeted rather than community-wide. In addition, the electronic search was restricted to PubMed/MEDLINE, Embase, and Scopus, so studies indexed exclusively in other databases may have been missing. Given the predominance of observational designs, methodological heterogeneity, and inconsistency across outcomes, the overall certainty of evidence for a causal relationship or a uniform T1DM-associated oral microbiome signature is low. The evidence is more appropriately interpreted as suggestive of associations between glycemic status and selected microbial findings, with substantial uncertainty regarding magnitude, direction, and generalizability. Future research should focus on well-designed prospective studies using standardized definitions of glycemic control, harmonized oral sampling by ecological niche, detailed characterization of periodontal and caries status, and prespecified adjustment for key confounders. Comprehensive sequencing and functional approaches may clarify whether reproducible community-level signatures exist and whether they have clinical utility. Any proposed microbial biomarker should undergo independent validation and demonstrate added value beyond established clinical and metabolic measures before clinical implementation.

## Conclusions

Overall, the available evidence indicates associations between glycemic control and selected oral microbial findings in T1DM, particularly in periodontal- and Candida-focused studies, but it does not establish a causal or uniform whole-community dysbiosis pathway. Interpretation is limited by cross-sectional designs, heterogeneous HbA1c definitions, variation in age and oral disease status, site-specific sampling, inconsistent confounder control, and the small number of comprehensive microbiome studies. Future longitudinal, clinically well-phenotyped, and community-level studies are required to determine whether reproducible microbiome signatures exist and whether they have diagnostic, prognostic, or therapeutic relevance. 

## Supplementary Information

Below is the link to the electronic supplementary material.

Supplementary Material 1.

Supplementary Material 2.

Supplementary Material 3.

## Data Availability

No datasets were generated or analysed during the current study.
