# Supplementary material for: Oral microbiome alterations in individuals with type 1 diabetes: a systematic review
Source: Clin Oral Investig. 2026 Oct 9;30(11):516. doi: 10.1007/s00784-026-07205-y (PMC13652932; doi:10.1007/s00784-026-07205-y)
Supplement: Supplementary file 1 [file 784_2026_7205_MOESM1_ESM.docx]

**Supplementary File**

- **PubMed**

**Population: #1: (((((((((((((((((diabetes mellitus[MeSH Terms]) OR (diabetes mellitus type 1 5[MeSH Terms])) OR (diabetes mellitus, insulin dependent[MeSH Terms])) OR (diabetes mellitus, insulin dependent, 1[MeSH Terms])) OR (insulin dependent diabetes mellitus[MeSH Terms])) OR (insulin dependent diabetes mellitus 1[MeSH Terms])) OR (type 1 diabetes[MeSH Terms])) OR (type 1 diabetes mellitus[MeSH Terms])) OR (diabetes mellitus)) OR (T1DM)) OR (T1D)) OR (IDDM)) OR (diabetic)) OR (autoimmune diabetes[MeSH Terms])) OR (lada, latent autoimmune diabetes in adults[MeSH Terms])) OR (latent autoimmune diabetes in adults[MeSH Terms])) OR (latent autoimmune diabetes of adults[MeSH Terms])) OR (diabetes)**

**Intervention: #2: ((((((((((((control, glycemic[MeSH Terms]) OR (glycemic control[MeSH Terms])) OR (glycemic index[MeSH Terms])) OR (glycated hemoglobin a1c[MeSH Terms])) OR (glycosylated hemoglobin a1c[MeSH Terms])) OR (hb a1c[MeSH Terms])) OR (hemoglobin a1c, glycated[MeSH Terms])) OR (hemoglobin a1c, glycosylated[MeSH Terms])) OR (glycohemoglobin[MeSH Terms])) OR (blood glucose[MeSH Terms])) OR (blood glucose control[MeSH Terms])) OR (control, blood glucose[MeSH Terms])) OR (glycaemic control)**

**Comparison: #3: -**

**Outcome: #4: (((((((((((((((((((((((saliva) OR (oral microbiota)) OR (oral microbiome)) OR (salivary microbiome)) OR (streptococc*)) OR (salivary microbiota)) OR (s. mutans)) OR (lactobacill*)) OR (oral microbiology)) OR (microbiological assessment)) OR (microbial oral health)) OR (salivary status)) OR (oral biofilm)) OR (cariogenic bacteria)) OR (microbiome)) OR (dysbiosis)) OR (cariogenic biofilm*)) OR (cariogenic pathogen*)) OR (oral microorganism*)) OR (oral microflora)) OR (acid producing bacteria)) OR (caries, dental[MeSH Terms])) OR (caries, cervical[MeSH Terms])) OR (caries, root[MeSH Terms])**

**#1 AND #2 AND #4: 2358 hits in Pubmed (14.06.2025)**

- **Embase:**

**('type 1 diabetes mellitus'/exp OR 'insulin dependent diabetes mellitus'/exp OR 'diabetes mellitus type 1':ti,ab OR 'type 1 diabetes':ti,ab OR T1DM:ti,ab OR T1D:ti,ab OR IDDM:ti,ab OR LADA:ti,ab OR 'latent autoimmune diabetes':ti,ab) AND ('glycemic control'/exp OR 'hemoglobin a1c'/exp OR 'blood glucose'/exp OR HbA1c:ti,ab OR 'glycaemic control':ti,ab OR 'poor glycemic control':ti,ab OR 'blood glucose':ti,ab OR 'glycosylated hemoglobin':ti,ab OR 'glycated hemoglobin':ti,ab) AND ('oral microbiome'/exp OR 'oral microbiota'/exp OR 'salivary microbiome':ti,ab OR 'oral microflora':ti,ab OR 'oral biofilm':ti,ab OR 'oral microbiology':ti,ab OR 'salivary microbiota':ti,ab OR streptococc*:ti,ab OR lactobacill*:ti,ab OR 's. mutans':ti,ab OR dysbiosis:ti,ab OR 'cariogenic bacteria':ti,ab OR 'oral bacteria':ti,ab OR 'acidogenic bacteria':ti,ab OR 'cariogenic pathogen*':ti,ab OR 'oral health'/exp)**

- **2824 results in Embase.com (11.06.2025)**
- **Scopus:**

**TITLE-ABS-KEY(**

**("type 1 diabetes mellitus" OR "insulin dependent diabetes mellitus" OR "diabetes mellitus type 1" OR T1DM OR T1D OR IDDM OR LADA OR "latent autoimmune diabetes")	AND	("glycemic control" OR HbA1c OR "hemoglobin A1c" OR "glycaemic control" OR "poor glycemic control" OR "blood glucose" OR "glycosylated hemoglobin" OR "glycated hemoglobin") AND ("oral microbiome" OR "oral microbiota" OR "salivary microbiome" OR "oral microflora" OR "oral biofilm" OR "oral microbiology" OR "salivary microbiota" OR streptococc* OR lactobacill* OR "s. mutans" OR dysbiosis OR "cariogenic bacteria" OR "oral bacteria" OR "acidogenic bacteria" OR "cariogenic pathogen*" OR "oral health"))**

- **1201 results (11.06.2025)**
