# Supplementary material for: Oral microbiome alterations in individuals with type 1 diabetes: a systematic review
Source: Clin Oral Investig. 2026 Oct 9;30(11):516. doi: 10.1007/s00784-026-07205-y (PMC13652932; doi:10.1007/s00784-026-07205-y)
Supplement: Supplementary file 3 [file 784_2026_7205_MOESM3_ESM.docx]

**Supplementary Table S1. Quantitative findings relating glycemic control to oral microbial and periodontal outcomes**

| **Study** | **Glycemic measure / comparison** | **Outcome** | **Quantitative finding** | **Statistical measure / interpretation** |
| --- | --- | --- | --- | --- |
| Jensen et al., 2021 [46] | Median HbA1c 8.5% (range 5.8–13.3%) | Plaque index | HbA1c positively correlated with plaque index | ρ = 0.34; p = 0.002 |
| Jensen et al., 2021 [46] | HbA1c | Gingival index | HbA1c positively correlated with gingival index | ρ = 0.30; p = 0.009 |
| Jensen et al., 2021 [46] | HbA1c | Bleeding on probing | HbA1c positively correlated with bleeding on probing | ρ = 0.44; p = 0.0001 |
| Jensen et al., 2021 [46] | Per 1% increase in HbA1c | Bleeding on probing | Average 25% increase in bleeding on probing | p = 0.002 |
| Jensen et al., 2021 [46] | Per 1% increase in HbA1c | Sites with probing depth >3 mm | 54% increase in the rate of sites with probing depth >3 mm | p = 0.003 |
| Jensen et al., 2021 [46] | Higher HbA1c | Gingival microbiota diversity | Higher phylogenetic alpha diversity and greater compositional variation; association was not observed in buccal microbiota | alpha diversity p = 0.008; beta diversity p = 0.02 |
| Schara et al., 2013 [54] | T. forsythia detected vs not detected | HbA1c | 7.5 ± 1.4% vs 6.8 ± 0.5% | p = 0.001 |
| Schara et al., 2013 [54] | T. denticola detected vs not detected | HbA1c | 7.5 ± 1.8% vs 7.0 ± 0.5% | p = 0.001 |
| Schara et al., 2013 [54] | 38 subgingival plaque samples | Pathogen prevalence | T. forsythia 48%; T. denticola 31%; P. gingivalis 26%; P. intermedia 9%; A. actinomycetemcomitans 7% | Descriptive prevalence |
| Pachoński et al., 2021 [45] | Poor control (HbA1c >7.5%), well control (≤7.5%), healthy controls | Total cultured microorganisms | 160 isolates in poor-control group; 158 in well-control group; 103 in healthy controls | Poor vs healthy p = 0.003; well vs healthy p < 0.001; poor vs well p = 0.195 |

*Note: Only numerical results that could be verified directly from the original publication were entered. Absence from this table should not be interpreted as evidence that a study reported no numerical data. Heterogeneous outcomes were not transformed or pooled.*

1
